# Supplementary material for: An intranasal subunit vaccine induces protective systemic and mucosal antibody immunity against respiratory viruses in mouse models
Source: Nat Commun. 2025 May 1;16:3999. doi: 10.1038/s41467-025-59353-6 (PMC12045997; doi:10.1038/s41467-025-59353-6)
Supplement: Supplementary file 1 — Supplementary Information [file 41467_2025_59353_MOESM1_ESM.pdf]

Supplementary Information for:

An intranasal subunit vaccine induces protective systemic and mucosal antibody immunity against respiratory viruses in mouse models

Authors: Aina Karen Anthi[1,2,3], Anette Kolderup[1,2,3]†, Eline Benno Vaage[1,3,4]†, Malin Bern[1,2]†, Sopisa Benjakul[1,2,3]‡, Elias Tjärnhage[1,4]‡, Fulgencio Ruso-Julve[1,2,3], Kjell-Rune Jensen[1,2,3], Heidrun Elisabeth Lode[1,2,3,5], Marina Vaysburd[6], Jeannette Nilsen[1,2,3], Marie Leangen Herigstad[1,2,3], Siri Aastedatter Sakya[1,2,3], Lisa Tietze[1,3], Diego Pilati[7], Mari Nyquist-Andersen[1,2,3], Mirjam Dürkoop[1,2,3], Torleif Tollefsrud Gjølberg[1,2,3,5], Linghang Peng[8], Stian Foss[1,2,3], Morten C. Moe[5], Benjamin E. Low[9], Michael V. Wiles[9], David Nemazee[8], Frode L. Jahnsen[4,10], John Torgils Vaage[1], Kenneth A. Howard[7], Inger Sandlie[11], Leo C. James[6], Gunnveig Grødeland[1,4], Fridtjof Lund-Johansen[1,3], and Jan Terje Andersen[1,2,3]*

Affiliations:

[1]Department of Immunology, Oslo University Hospital Rikshospitalet; 0372 Oslo, Norway.

[2]Institute of Clinical Medicine and Department of Pharmacology, University of Oslo and Oslo University Hospital Rikshospitalet; 0372 Oslo, Norway.

[3]Precision Immunotherapy Alliance (PRIMA), University of Oslo; 0372 Oslo, Norway.

[4]Institute of Clinical Medicine, University of Oslo; 0372 Oslo, Norway.

[5]Center of Eye Research, Department of Ophthalmology, Oslo University Hospital Ullevål and University of Oslo; 0450 Oslo, Norway.

[6]Protein and Nucleic Acid Chemistry Division, Medical Research Council, Laboratory of Molecular Biology; Cambridge CB2 0QH, United Kingdom.

[7]Interdisciplinary Nanoscience Center (iNANO), Department of Molecular Biology and Genetics, Aarhus University; DK-8000 Aarhus C, Denmark.

[8]Department of Immunology and Microbiology, The Scripps Research Institute, La Jolla; CA 92037, USA.

[9]The Jackson Laboratory, Bar Harbor; ME 04609, USA.

[10]Department of Pathology, Oslo University Hospital Rikshospitalet; 0372 Oslo, Norway.

[11]Department of Biosciences, University of Oslo; 0371 Oslo, Norway.

† These authors contributed equally to this work

‡ These authors contributed equally to this work

*Corresponding author. Email: j.t.andersen@medisin.uio.no

Supplementary Information List:

Supplementary Figure 1: SDS-PAGE and binding properties of recombinant RBD and RBD-fused MSA.
Supplementary Figure 2: Intranasal vaccination with RBD-fused MSA induces RBD-specific antibody responses.
Supplementary Figure 3: Intranasal vaccination with RBD-fused MSA induces RBD-specific antibody responses at least as robust as following intramuscular vaccination with an mRNA-based vaccine.
Supplementary Figure 4: Gating strategy for flow analysis of antigen-specific germinal center B-cells.
Supplementary Figure 5: Characterization of RBD-fused human albumin variants.
Supplementary Figure 6: Intranasal vaccination with RBD-fused QMP induces RBD-specific antibody responses.
Supplementary Figure 7: Characterization of RBD-fused Tf.
Supplementary Figure 8: Characterization of HA-fused QMP and Tf.
Supplementary Figure 9: Characterization of HA-fused MSA variants.
Supplementary Figure 10: HA-specific antibody responses induced following intranasal vaccination with HA-fused MSA variants in female BALB/c mice.
Supplementary Figure 11: Intranasal vaccination with albumin fusions is dependent on adjuvant to induce antigen-specific antibody responses.
Supplementary Figure 12: Amino acid sequence alignment of mouse and human albumin.
Supplementary Figure 13: Intranasal vaccination with RBD-fused human albumin induces RBD-specific antibody responses in mice expressing both human albumin and human FcRn (HSA/hFcRn mice).
Supplementary Figure 14: Intranasal vaccination with antigen-fused MSA does not induce antibodies against MSA in female BALB/c mice.
Supplementary Figure 15: Structural comparison of amino acid substitutions in a selected panel of RBD variants derived from SARS-CoV-2 strains.
Supplementary Figure 16: Intranasal vaccination with Wuhan RBD-fused albumin induces antibody responses against a diverse set of RBD variants.
Supplementary Figure 17: Characterization of mouse IgA in samples collected post intranasal vaccination in mice.

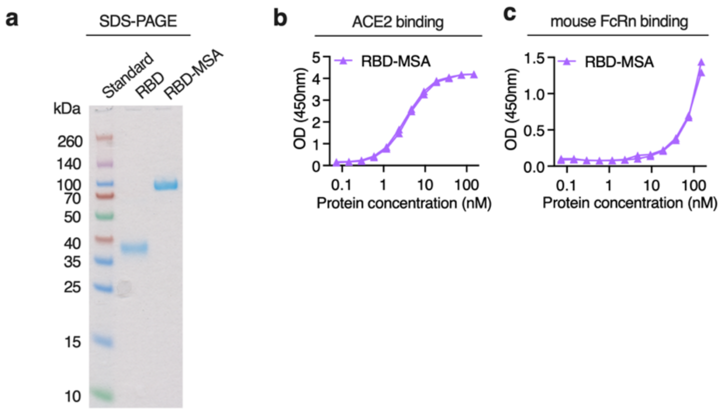

Supplementary Fig. 1: SDS-PAGE and binding properties of recombinant RBD and RBD-fused MSA. (a) Non-reducing SDS-PAGE of purified fractions of RBD and RBD-MSA, representative from three independent experiments. (**b-c**) ELISA binding of RBD-MSA to (b) human ACE2 and (c) mouse FcRn at pH 5.5. Curve plots are presented as each replicate of technical duplicates.

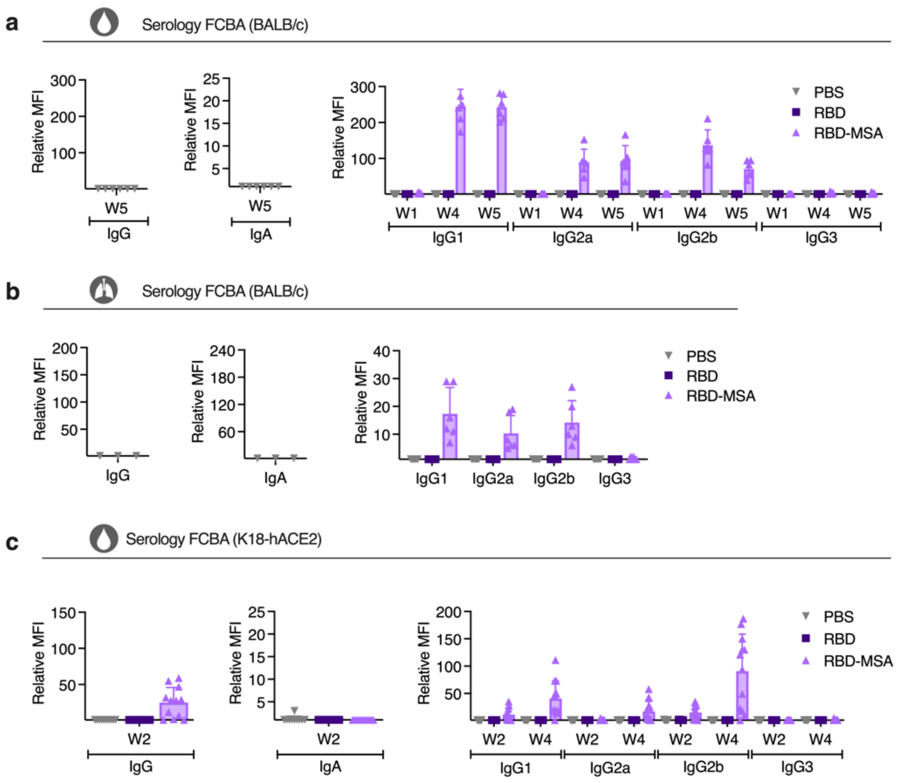

Supplementary Fig. 2: Intranasal vaccination with RBD-fused MSA induces RBD-specific antibody responses. (**a-b**) RBD-specific mouse IgG, IgA, IgG1, IgG2a, IgG2b and IgG3 responses detected (a) in serum samples at week 1, 4 and 5, and (b) BALF samples at endpoint, post intranasal vaccination of female BALB/c mice with equimolar amounts of RBD and RBD-MSA (prime dose: 6.2 µg and 19.9 µg, respectively) in combination with 20 µg CpG, or given PBS. **(c)** RBD-specific mouse IgG, IgA, IgG1, IgG2a, IgG2b and IgG3 responses detected in serum samples at week 2 and 4 post intranasal vaccination of female K18-ACE2 mice with equimolar amounts of RBD and RBD-MSA (prime dose: 6.2 µg and 19.9 µg, respectively) in combination with 20 µg CpG, or given PBS. Data analyzed by FCBA and presented as bar plots, which indicate group mean ± SD with individual mice represented as a single datapoint ((a) n=6, (b) IgG and IgA: PBS and RBD n=3 and RBD-MSA n=4, IgG subclasses: PBS and RBD-MSA n=6 and RBD n=4, and (c) PBS n=10 and RBD and RBD-MSA n=12). (a-c) Partially created in BioRender. Anthi, A. (2025) https://BioRender.com/ikhc1he.

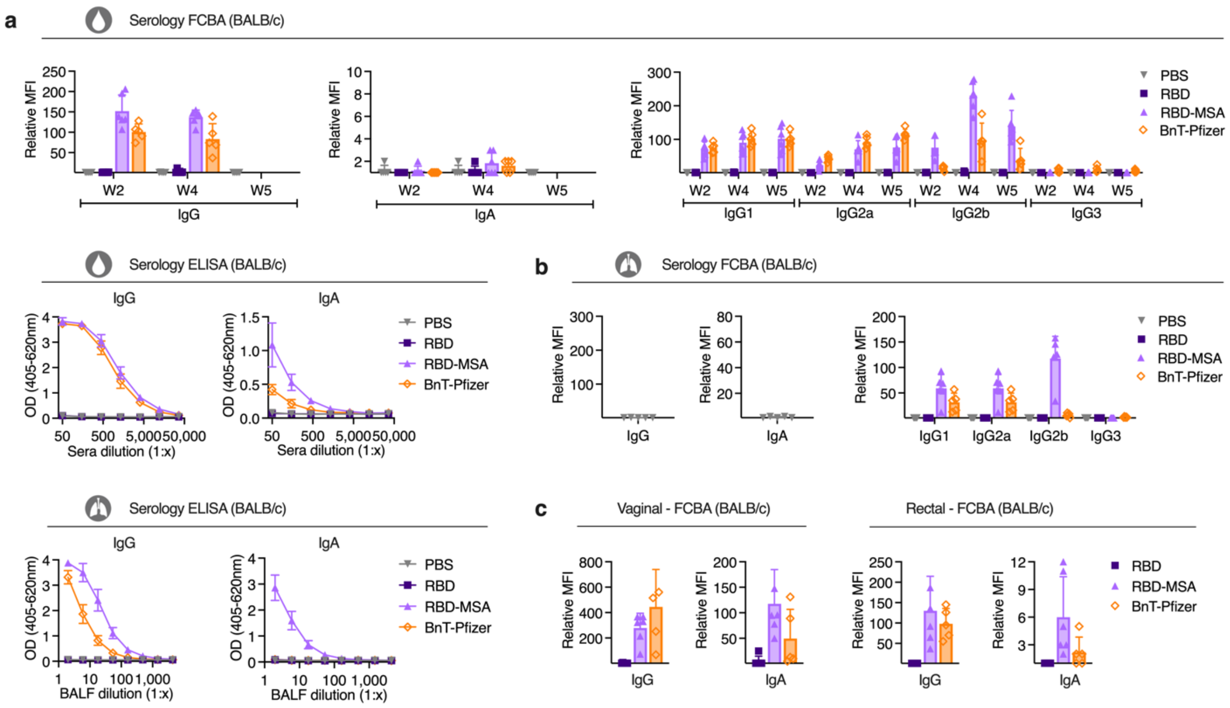

Supplementary Fig. 3: Intranasal vaccination with RBD-fused MSA induces RBD-specific antibody responses at least as robust as following intramuscular vaccination with an mRNA-based vaccine. **(a-c)** RBD-specific antibody responses of female BALB/c mice intranasally vaccinated with 0.22 nM RBD-MSA or RBD (prime dose: 6.2 µg and 19.9 µg, respectively) together with 20 µg CpG, or given PBS, or intramuscularly vaccinated with the mRNA vaccine from BioNTech-Pfizer (Comirnaty/BNT162b2) (prime dose: 3 µg). RBD-specific IgG, IgA, IgG1, IgG2a, IgG2b and IgG3 detected in (a) serum samples as week 2, 4 and 5, and (b) BALF samples at endpoint. (c) RBD-specific IgG and IgA detected in samples collected from the vaginal and rectal tracts at endpoint. Data analyzed by FCBA and presented as bar plots, which indicate group mean ± SD with individual mice represented as a single datapoint or by ELISA and presented as OD values with biological group mean ± SD (PBS and BnT-Pfizer n=5 and RBD and RBD-MSA n=6, or for BALF in ELISA n=5). (a and b) Partially created in BioRender. Anthi, A. (2025) https://BioRender.com/ikhc1he.

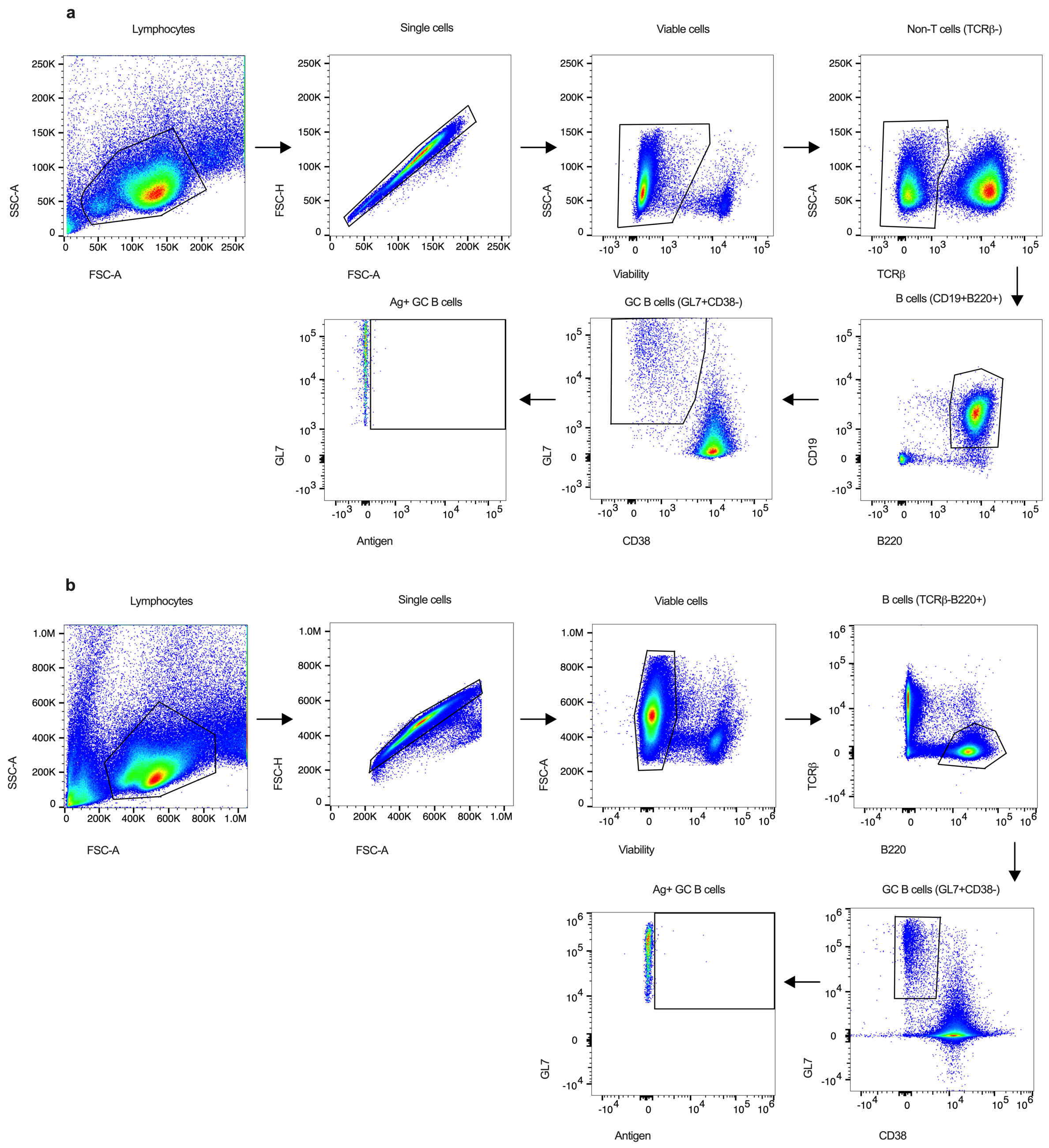

Supplementary Fig. 4: Gating strategy for flow analysis of antigen-specific germinal center B-cells. Mediastinal lymph nodes of intranasally vaccinated mice, or inguinal lymph nodes from PBS mice or intramuscularly vaccinated mice were harvested at endpoint and analyzed by flow cytometry using the representative gating strategy in (a) to determine antigen-specific germinal center B-cells (TCRβ$^-$B220$^+$CD19$^+$CD38$^-$GL7$^+$Ag$^+$), using markers specified in methods section. This gating strategy was used for analysis on FACSymphony A5 for the B-cell data shown in Fig. 3d and Fig. 4d. The representative gating strategy in (b) was used to determine antigen-specific germinal center B-cells (TCRβ-B220+CD38-GL7+Ag+) in Fig. 5c, which was analyzed on an Attune NxT Flow Cytometer.

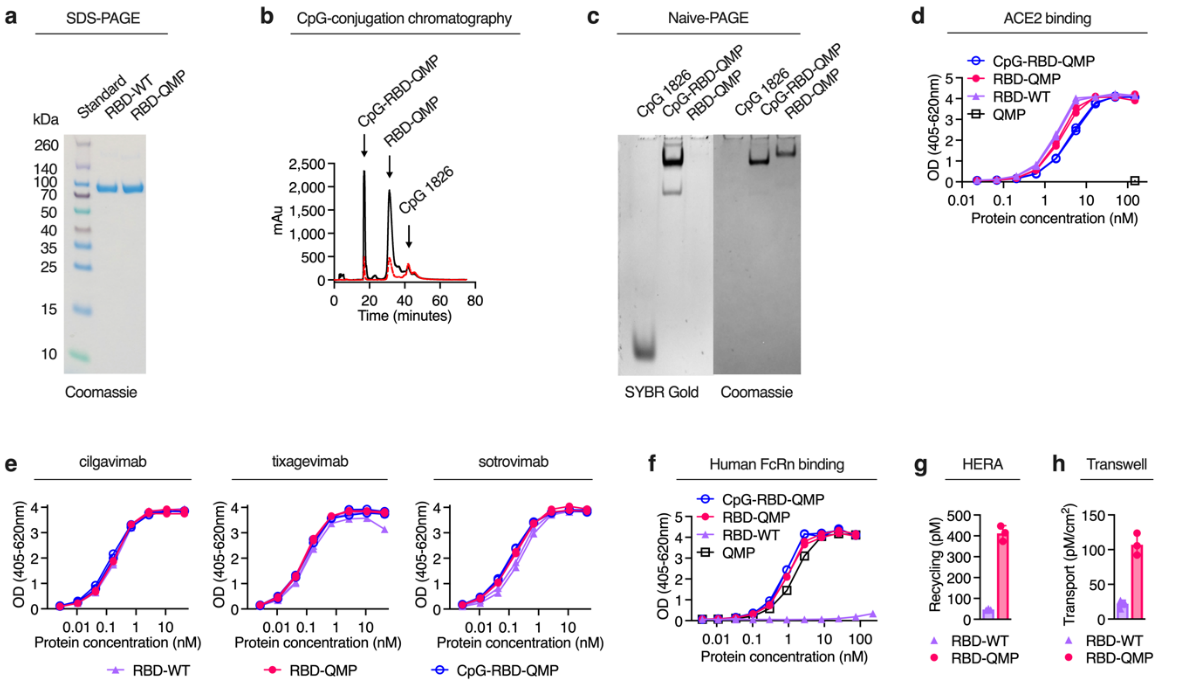

Supplementary Fig. 5: Characterization of RBD-fused human albumin variants. (a) Non-reducing SDS-PAGE analysis of RBD-WT and RBD-QMP, representative from three independent experiments. (**b**) Chromatogram from purification of RBD-fused albumin with CpG site-specifically conjugated to cysteine 34 (C34) (CpG-RBD-QMP), and (**c**) naive-PAGE analysis confirming the purity of the isolated CpG-RBD-QMP fraction displaying DNA by SYBR Gold and monomeric protein fraction by Coomassie staining, representative from three independent experiments. (**d**) ELISA binding of QMP and RBD-albumin fusion variants to human ACE2. (**e**) ELISA results showing binding of the commercial monoclonal IgG antibodies cilgavimab, tixagevimab and sotrovimab to the RBD-fused albumin fusion variants. (**f**) ELISA results showing human FcRn binding to QMP and the RBD-albumin fusion variants at pH5.5. (**g**) Human FcRn-mediated cellular rescue from intracellular degradation for RBD-WT and RBD-QMP in a human endothelial cell-based recycling assay (HERA). **(h)** Human FcRn-mediated apical to basolateral transcytosis of RBD-WT and RBD-QMP across polarized MDCK cells over-expressing the receptor. Curve plots are presented as each replicate of technical duplicates, bar plots are presented as technical triplicates (g), and technical triplicates or quadruplets (h) from one representative experiment, with the bar indicating group mean ± SD with each datapoint presented as single points.

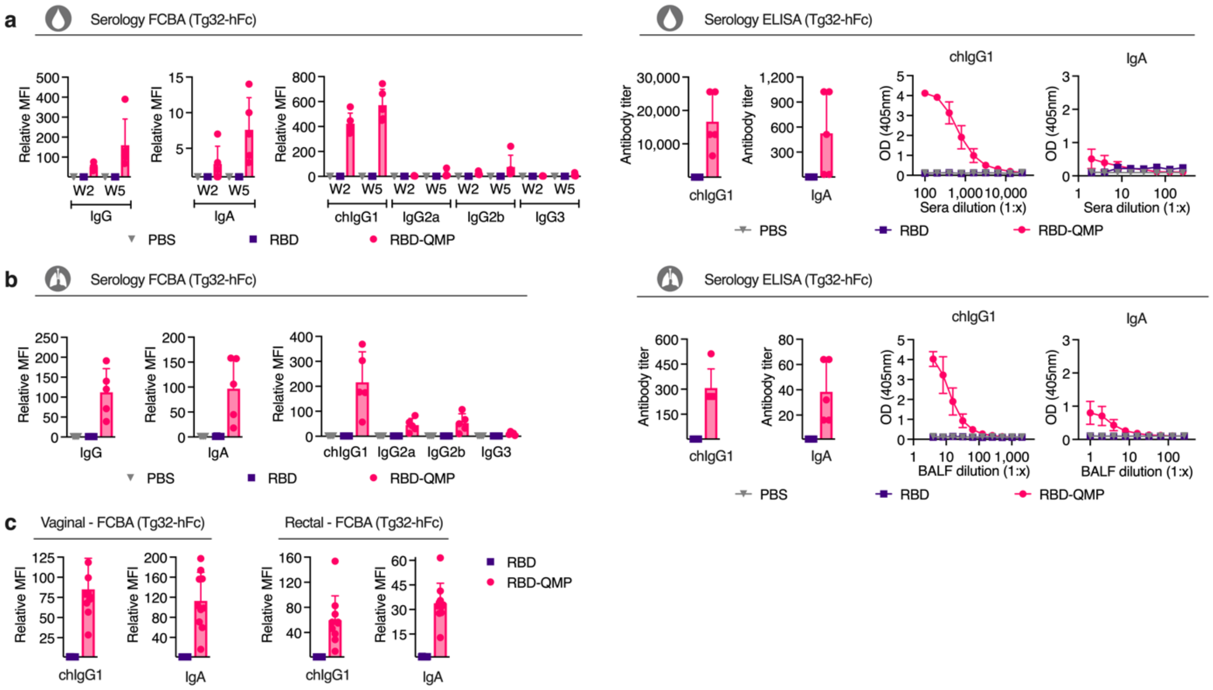

Supplementary Fig. 6: Intranasal vaccination with RBD-fused QMP induces RBD-specific antibody responses. (**a-c**) RBD-specific antibody responses in female Tg32-hFc mice intranasally vaccinated with RBD or RBD-QMP (prime dose: 6.2 µg and 20.0 µg, respectively) together with 20 µg CpG, or given PBS, measured as RBD-specific IgG, IgA, chimeric human IgG1 (chIgG1), IgG2a, IgG2b and IgG3 in (a) serum samples at weeks 2 and 5, and (b) BALF samples at endpoint, and as (c) chIgG1 and IgA in samples collected form the vaginal and rectal tracts. Data analyzed by FCBA or ELISA and presented as bar plots, which indicate group mean ± SD with individual mice represented as a single datapoint or by ELISA and presented as OD values with biological group mean ± SD ((a-b) n=5 and (c) RBD n=5 and RBD-QMP n=6). The results in (a-b) are from a repeated experiment independent of the results presented in Figure 2e. (a and b) Partially created in BioRender. Anthi, A. (2025) https://BioRender.com/ikhc1he.

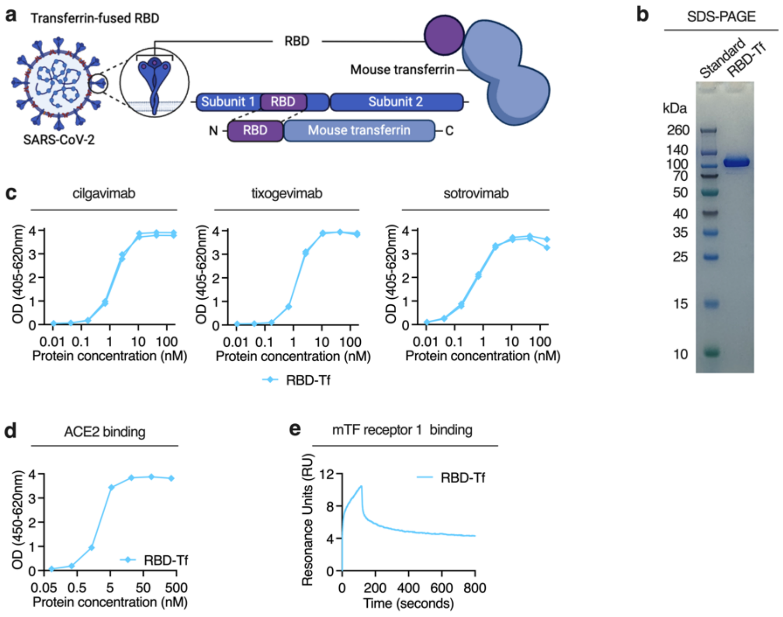

Supplementary Fig. 7: Characterization of RBD-fused Tf. (**a**) Illustration of a fusion design with RBD genetically fused to Tf. (**b**) Non-reducing SDS-PAGE analysis of purified RBD-Tf, representative from three independent experiments. (**c**) ELISA results showing binding of RBD-Tf to the commercial antibodies cilgavimab, tixagevimab and sotrovimab. (**d**) ELISA binding of RBD-Tf to human ACE2 and (**e**) SPR result showing binding of 8,000 nM RBD-Tf injected over immobilized mouse Transferrin receptor 1 (mTfR) (pH 5.5). The ELISA curve plots are presented as each replicate of technical duplicates, and the SPR plot is one representative curve of a technical triplicate. (a) Created in BioRender. Anthi, A. (2025) https://BioRender.com/ikhc1he.

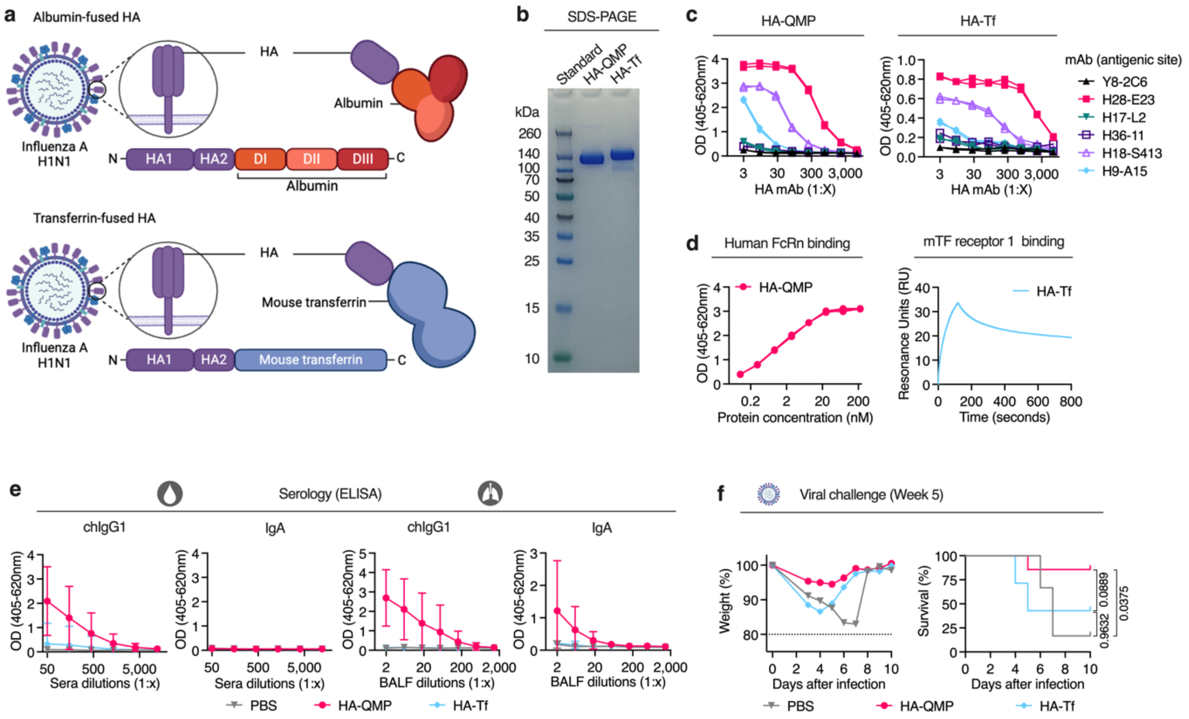

Supplementary Fig. 8: Characterization of HA-fused QMP and Tf. (**a**) Illustration of a fusion design with Influenza A H1N1 hemagglutinin (HA) (aa 18-519) fused to human albumin (top panel), or Tf (bottom panel). (**b**) Non-reducing SDS-PAGE of the respective purified HA fusions, representative from three independent experiments. (**c**) ELISA results for detection of epitope availability of HA fusions using epitope-specific monoclonal antibodies targeting the globular domain. (**d**) ELISA result showing binding of HA-QMP to the human FcRn receptor (pH 5.5) and SPR result showing binding of 8,000 nM HA-Tf over immobilized mouse TfR (pH 5.5). (**e**) ELISA showing HA-specific antibody responses in female and male Tg32-hFc mice intranasally vaccinated with HA-QMP or HA-Tf (prime dose: 26.9 µg and 28.9 µg, respectively) together with 20 µg CpG, or given PBS, measured as chIgG1 and IgA in serum and BALF samples. (**f**) Weight change following challenge with 5xLD50 influenza A H1N1 PR8 five weeks after prime of female mice. ELISA curve plots are presented (c and d) as each replicate of technical duplicates, or (e) as biological group mean ± SD (sera chIgG1: PBS n=7 (2M, 6F), HA-QMP n=12 (5M, 7F), HA-Tf n=13 (6M, 7F), sera IgA and BALF chIgG1 and IgA: PBS n=2, HA-QMP n=5, HA-Tf n=6 (all male)). SPR plot is a representative curve of a technical triplicate. Weight in (f) presented as percentage weight compared to the weight at day of infection as biological group mean ± SEM (PBS n=6 and HA-Tf and HA-QMP n=7 (all female)), and log-ranked Mantel-Cox test performed for survival. (a) Created or (e and f) partially created in BioRender. Anthi, A. (2025) https://BioRender.com/ikhc1he.

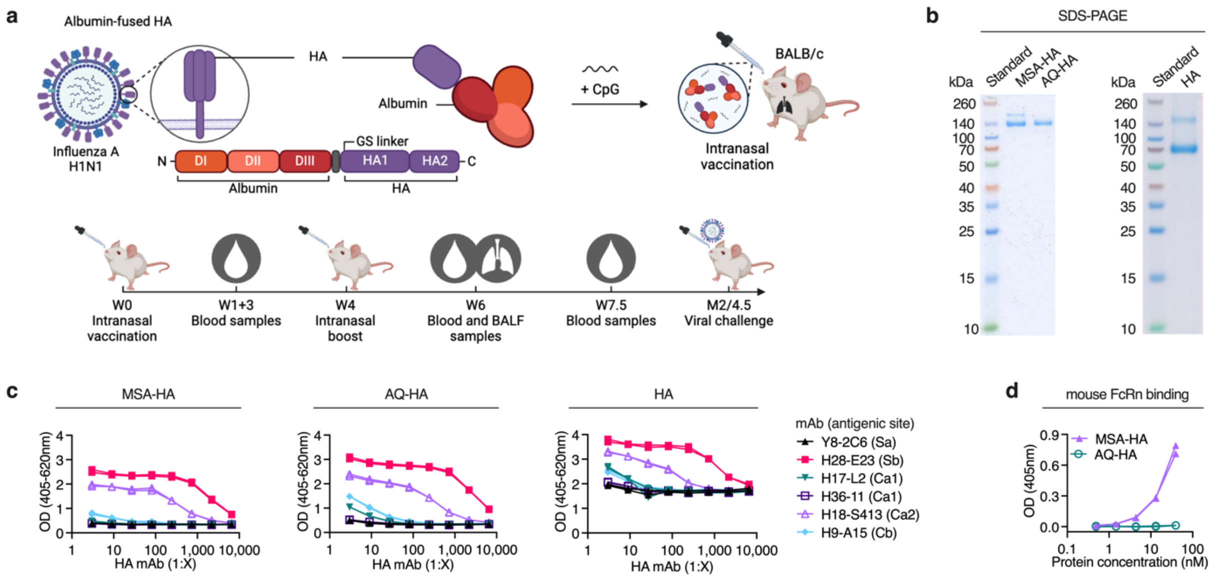

Supplementary Fig. 9: Characterization of HA-fused MSA variants. (**a**) Illustration of the fusion design where HA (aa 18-541) is genetically fused to MSA via a GS-linker (top panel), and the intranasal vaccination regimen for HA or HA-fused MSA mixed with CpG as adjuvant, or NaCl in BALB/c mice (bottom panel). (**b**) Non-reducing SDS-PAGE analysis of the HA-fused MSA variants (WT and AQ) and HA, representative from three independent experiments. (**c**) ELISA results for detection of epitope availability of HA or HA-fused MSA variants using epitope-specific monoclonal antibodies targeting the globular domain. (**d**) ELISA results showing binding of HA-MSA fusions (WT and AQ) to mouse FcRn at pH 5.5. Curve plots are presented as each replicate of technical duplicates. (a) Created in BioRender. Anthi, A. (2025) https://BioRender.com/ikhc1he.

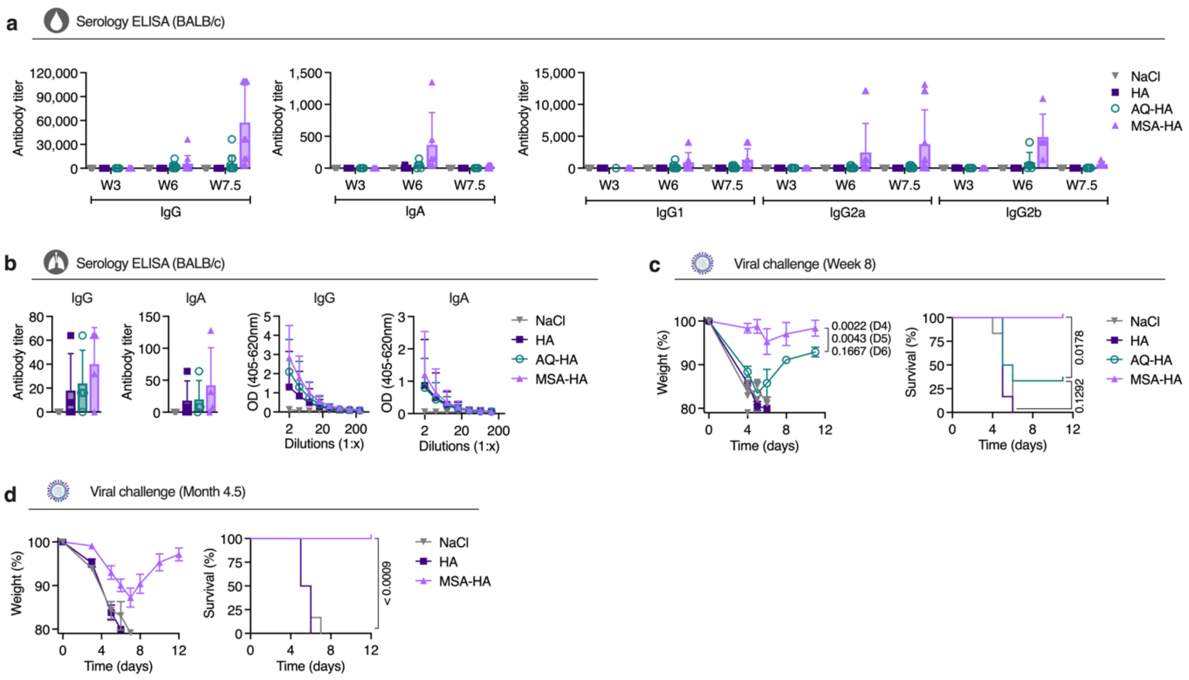

Supplementary Fig. 10: HA-specific antibody responses induced following intranasal vaccination with HA-fused MSA variants in female BALB/c mice. (**a**) HA-specific IgG, IgA, IgG1, IgG2a and IgG2b ELISA responses in serum samples at weeks 3, 5 and 7.5 after intranasal vaccination with equimolar amounts of MSA-HA, AQ-HA or unfused HA (prime dose: 27.5 µg, 27.5 µg and 13.1 µg, respectively), together with 20 µg CpG, or NaCl. (**b**) HA-specific IgG and IgA in BALF samples 6 weeks post intranasal vaccination, represented as antibody titer or OD values. Data analyzed by ELISA and presented as bar plots, which indicate group mean ± SD with individual mice represented as a single datapoint, or as curve plots with biological group mean ± SD ((a) IgG: n=12, IgA: n=6 (NaCl n=5), IgG1 and IgG2a: n=12, IgG2b: NaCl and HA n=5, AQ-HA=6, MSA-HA W3 and 6 n=6, W7.5 n=5, and (b) n=4). (**c-d**) Weight change and survival after intranasally vaccinated BALB/c mice were challenged with 5xLD50 influenza A H1N1 PR8, at (c) 8 weeks and (d) 4.5 months after initial vaccination. (c-d) Weight presented as percentage weight compared to the weight at day of infection, from 6 mice per group, and log-ranked Mantel-Cox test performed for survival. (a-d) Partially created in BioRender. Anthi, A. (2025) https://BioRender.com/ikhc1he.

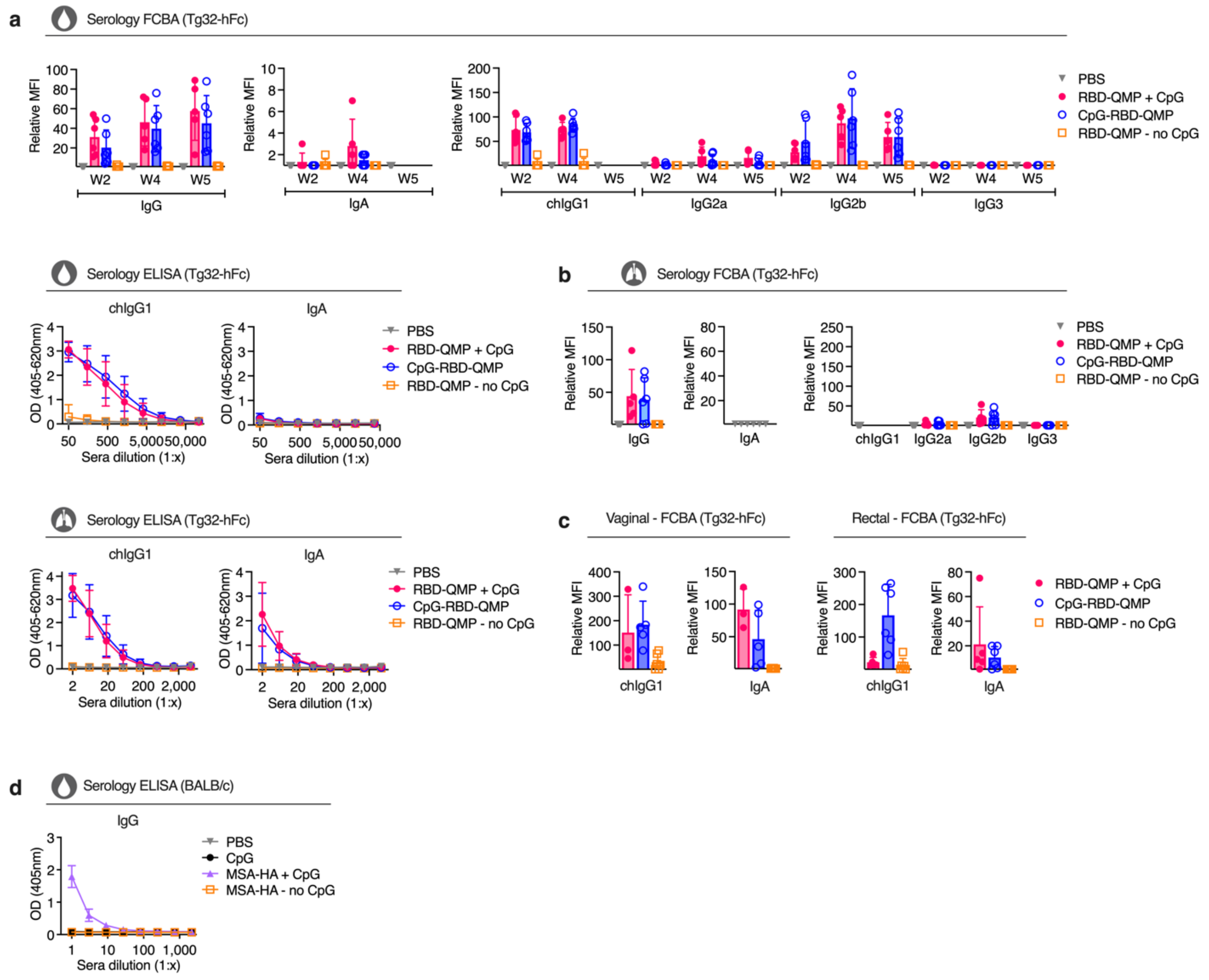

Supplementary Fig. 11: Intranasal vaccination with albumin fusions is dependent on adjuvant to induce antigen-specific antibody responses. (**a-c**) RBD-specific antibody responses in female Tg32-hFc mice intranasally vaccinated with RBD-QMP together with 20 µg CpG, without CpG (prime dose: 20.0 µg), or directly conjugated to CpG (prime dose: 21.4 µg), or given PBS, detected using IgG, IgA, chIgG1, IgG2a, IgG2b and IgG3 in (a) serum samples at week 2, 4 and 5, and (b) BALF samples at endpoint, and as (c) chIgG1 and IgA in samples collected from the vaginal and rectal tracts. (**d**) HA-specific IgG responses in sera at week 4 of female BALB/c mice intranasally vaccinated with MSA-HA together with 20 µg CpG or without CpG (prime dose: 27.5 µg), or given 20 µg CpG or PBS. Data analyzed by FCBA and presented as bar plots, which indicate group mean ± SD with individual mice represented as a single datapoint, or by ELISA and presented as OD values with biological group mean ± SD (n=4-6, except (c) vaginal RBD-QMP + CpG n=3). (a) n=6, except RBD-QMP + CpG n=5 for IgG, IgA, IgG subclasses W4 and W5, (b) n=6 except RBD-QMP + CpG n=5 (and n= 4 in IgG ELISA), (c) vaginal: RBD-QMP + CpG, PBS, CpG-RBD-QMP, RBD-QMP no CpG, n=3,4,5,6, respectively. Rectal: RBD-QMP + CpG n=5 and PBS, CpG-RBD-QMP, RBD-QMP no CpG n=6, except IgG PBS n=5, and (d) n=6. (a,b,d) Partially created in BioRender. Anthi, A. (2025) https://BioRender.com/ikhc1he.

```
MSA       1   EAHKSEIAHRYNDLGEQHFKGLVLIAFSQYLQKCSYDEHAKLVQEVTDFA    50
HSA       1   DAHKSEVAHRFKDLGEENFKALVLIAFAQYLQQCPFEDHVKLVNEVTEFA    50

MSA      51   KTCVADESAANCDKSLHTLFGDKLCAIPNLRENYGELADCCTKQEPERNE   100
HSA      51   KTCVADESAENCDKSLHTLFGDKLCTVATLRETYGEMADCCAKQEPERNE   100

MSA     101   CFLQHKDDNPSLPPFERPEAEAMCTSFKENPTTFMGHYLHEVARRHPYFY   150
HSA     101   CFLQHKDDNPNLPRLVRPEVDVMCTAFHDNEETFLKKYLYEIARRHPYFY   150

MSA     151   APELLYYAEQYNEILTQCCAEADKESCLTPKLDGVKEKALVSSVRQRMKC   200
HSA     151   APELLFFAKRYKAAFTECCQAADKAACLLPKLDELRDEGKASSAKQRLKC   200

MSA     201   SSMQKFGERAFKAWAVARLSQTFPNADFAEITKLATDLTKVNKECCHGDL   250
HSA     201   ASLQKFGERAFKAWAVARLSQRFPKAEFAEVSKLVTDLTKVHTECCHGDL   250

MSA     251   LECADDRAELAKYMCENQATISSKLQTCCDKPLLKKAHCLSEVEHDTMPA   300
HSA     251   LECADDRADLAKYICENQDSISSKLKECCEKPLLEKSHCIAEVENDEMPA   300

MSA     301   DLPAIAADFVEDQEVCKNYAEAKDVFLGTFLYEYSRRHPDYSVSLLLRLA   350
HSA     301   DLPSLAADFVESKDVCKNYAEAKDVFLGMFLYEYARRHPDYSVVLLLRLA   350

MSA     351   KKYEATLEKCCAEANPPACYGTVLAEFQPLVEEPKNLVKTNCDLYEKLGE   400
HSA     351   KTYETTLEKCCAAADPHECYAKVFDEFKPLVEEPQNLIKQNCELFEQLGE   400

MSA     401   YGFQNAILVRYTQKAPQVSTPTLVEAARNLGRVGTKCCTLPEDQRLPCVE   450
HSA     401   YKFQNALLVRYTKKVPQVSTPTLVEVSRNLGKVGSKCCKHPEAKRMPCAE   450

MSA     451   DYLSAILNRVCLLHEKTPVSEHVTKCCSGSLVERRPCFSALTVDETYVPK   500
HSA     451   DYLSVVLNQLCVLHEKTPVSDRVTKCCTESLVNRRPCFSALEVDETYVPK   500

MSA     501   EFKAETFTFHSDICTLPEKEKQIKKQTALAELVKHKPKATAEQLKTVMDD   550
HSA     501   EFNAETFTFHADICTLSEKERQIKKQTALVELVKHKPKATKEQLKAVMDD   550

MSA     551   FAQFLDTCCKAADKDTCFSTEGPNLVTRCKDALA-   584
HSA     551   FAAFVEKCCKADDKETCFAEEGKKLVAASQAALGL   585
```

Supplementary Fig. 12: Amino acid sequence alignment of mouse and human albumin. Sequence alignment displaying amino acid differences highlighted in red color between the albumin derived from mouse (MSA) or human (HSA). Sequence identity: 422/585 (72.1%). Made by using EMBOSS Needle Pairwise alignment with the following NCBI accession numbers: MSA: AAH49971.1, HSA: AAA98797.1.

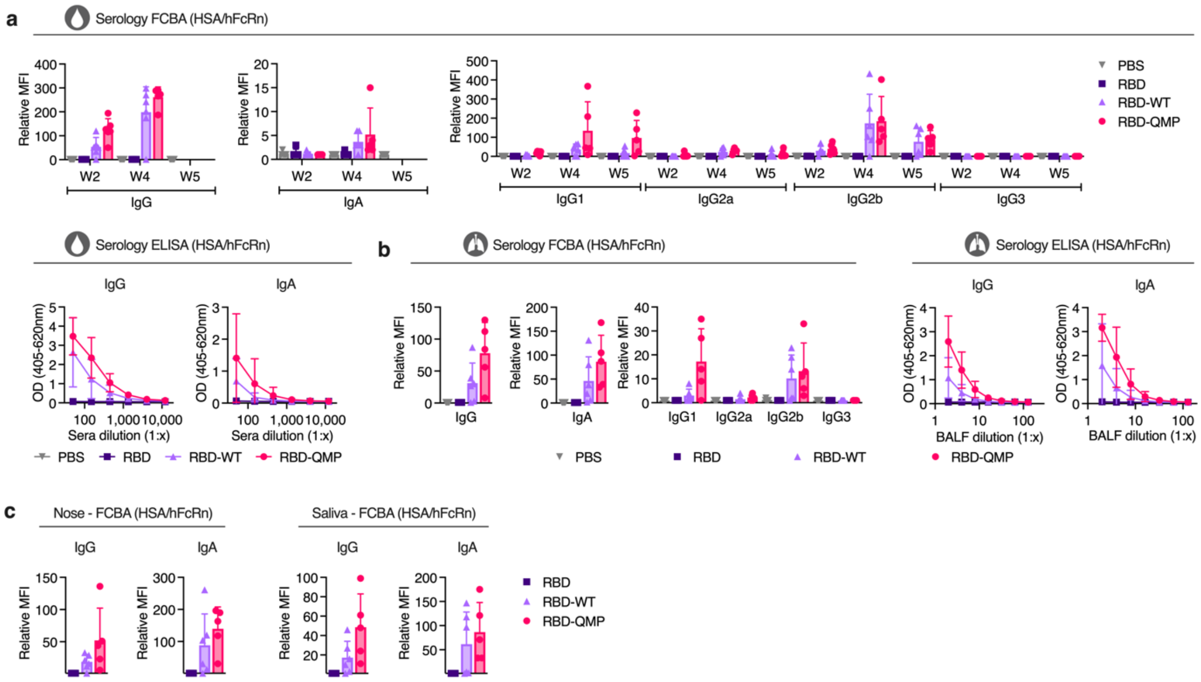

Supplementary Fig. 13: Intranasal vaccination with RBD-fused human albumin induces RBD-specific antibody responses in mice expressing both human albumin and human FcRn (HSA/hFcRn mice). (**a-c**) RBD-specific IgG, IgA, IgG1, IgG2a, IgG2b and IgG3 responses in female HSA/hFcRn mice following intranasal vaccination with RBD, RBD-WT, RBD-QMP (prime dose: 6.2 µg, 20.0 µg and 20.0 µg, respectively) together with 20 µg CpG, or given PBS, in (a) serum samples at week 2, 4, and 5, and (b) BALF samples at endpoint, and (c) RBD-specific IgG and IgA responses in samples collected from the upper respiratory tract at endpoint. Data analyzed by FCBA and presented as bar plots, which indicate group mean ± SD with individual mice represented as a single datapoint, or by ELISA and presented as OD values with biological group mean ± SD (n=5-6). (a) n=6, except RBD-QMP (not subclasses W2) and PBS IgG1 W2 n=5, (b) n=6, except RBD-QMP n=5, and RBD-WT in ELISA n=5, (c) n=6, except RBD-QMP n=5. (a and b) Partially created in BioRender. Anthi, A. (2025) https://BioRender.com/ikhc1he.

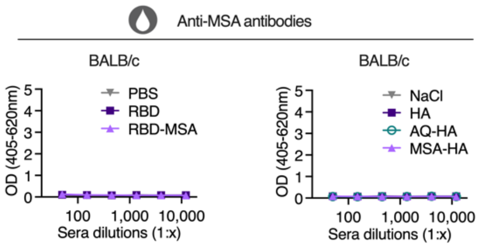

Supplementary Fig. 14: Intranasal vaccination with antigen-fused MSA does not induce antibodies against MSA in female BALB/c mice. ELISA results showing no detectable MSA-specific mouse IgG antibodies in samples collected from female BALB/c mice at endpoint post intranasal vaccination with antigen (prime dose: RBD 6.2 µg, and HA 13.1 µg) or MSA-fusions (prime dose: RBD-MSA 19.9 µg, AQ-HA 27.5 µg and MSA-HA 27.5 µg) together with 20 µg CpG, or given PBS/NaCl, presented as OD values with biological group mean ± SD (left curve n=6, right curve n=12). Partially created in BioRender. Anthi, A. (2025) https://BioRender.com/ikhc1he.

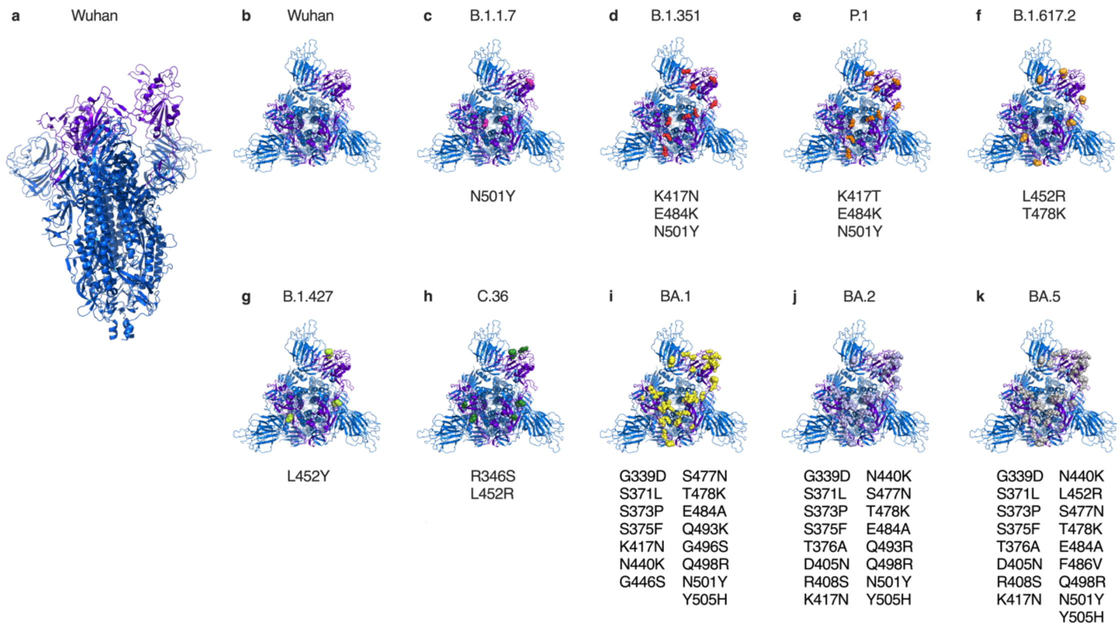

Supplementary Figure 15: Structural comparison of amino acid substitutions in a selected panel of RBD variants derived from SARS-CoV-2 strains. (**a-b**) Structural illustrations of trimers of spike (blue) with RBD (purple) from SARS-CoV-2 Wuhan as (a) side view and (b) top view. (**c-k**) Top view of (c) B.1.1.7 (Alpha), (d) B.1.351 (Beta), (e) P.1 (Gamma), (f) B.1.617.2 (Delta), (g) B.1.427, (h) C.36, (i) BA.1 (Omicron), (j) BA.2 (Omicron), and (k) BA.5 (Omicron) variants of SARS-CoV-2, containing indicated amino acid mutations in RBD. (a-k) Generated using PyMOL from PDB: 7DDN, and illustrated using GraphPad Prism.

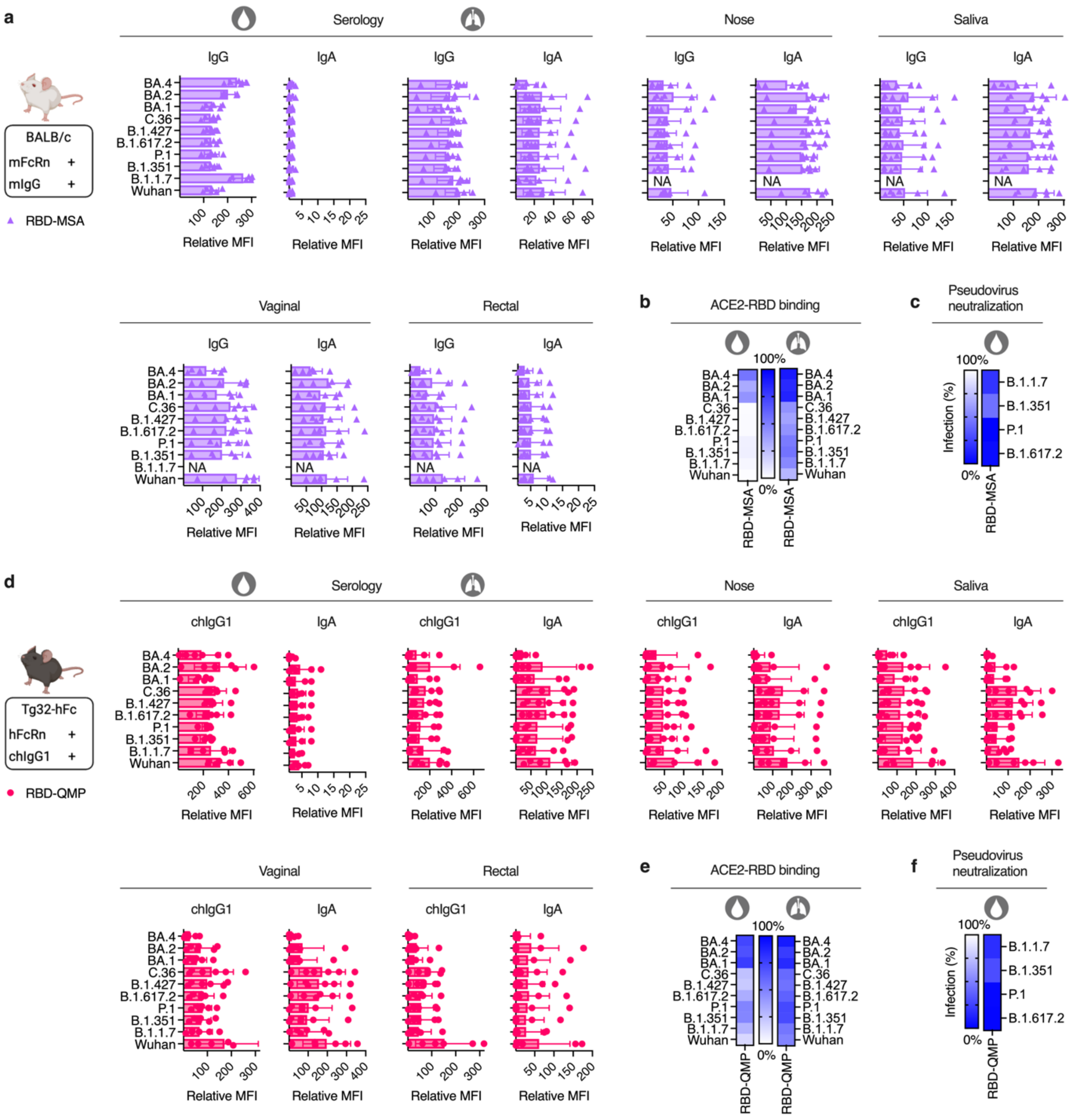

Supplementary Fig. 16: Intranasal vaccination with Wuhan RBD-fused albumin induces antibody responses against a diverse set of RBD variants. (a and d) RBD variant-specific IgG and IgA antibody responses in in serum, BALF, nose, saliva, vaginal tract and rectal tract samples collected from (a) female BALB/c and (d) female Tg32-hFc mice at endpoint following intranasal vaccination with RBD-fused albumin based on RBD Wuhan (prime dose: RBD-MSA 19.9 µg and RBD-QMP 20.0 µg, together with 20 µg CpG). (**b** and **e**) The ability of the antibodies in serum and BALF samples, at endpoint post intranasal vaccination of (b) BALB/c mice and (e) Tg32-hFc mice, to inhibit human ACE2 binding to the RBD variants. (**c** and **f**) The ability of the RBD-specific antibodies in serum samples at endpoint following intranasal vaccination of (c) BALB/c mice and (f) Tg32-hFc mice to block cellular infection by SARS-CoV-2 variant pseudoviruses in a pseudovirus neutralization assay. Data analyzed by FCBA and presented as bar plots, which indicate group mean ± SD with individual mice represented as a single datapoint (n=6), or as heatmap with the biological mean of pooled sera within each group run in technical duplicates (n=6).

NA = not analyzed. (a-f) Partially created in BioRender. Anthi, A. (2025) https://BioRender.com/ikhc1he.

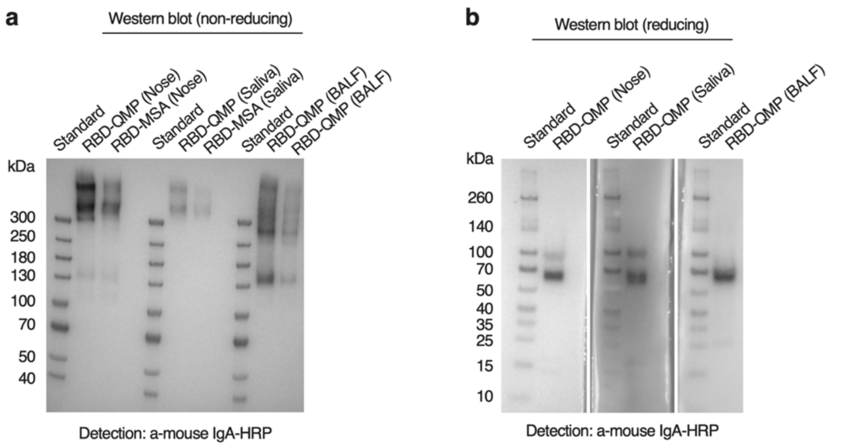

Supplementary Fig. 17: Characterization of mouse IgA in samples collected post intranasal vaccination in mice. Western blot analysis of mouse IgA present in nose, saliva and BALF samples at endpoint post intranasal vaccination of female mice with RBD-QMP and RBD-MSA (prime dose: 20.0 μg RBD-QMP and 19.9 μg RBD-MSA, together with 20 μg CpG) under (**a**) non-reducing and (**b**) reducing conditions. Samples within each treatment group were pooled. Representative blots from three independent experiments (BALF, non-reducing) or one independent experiment.
