# Supplementary material for: An intranasal subunit vaccine induces protective systemic and mucosal antibody immunity against respiratory viruses in mouse models
Source: Nat Commun. 2025 May 1;16:3999. doi: 10.1038/s41467-025-59353-6 (PMC12045997; doi:10.1038/s41467-025-59353-6)
Supplement: Supplementary file 2 — Reporting Summary [file 41467_2025_59353_MOESM2_ESM.pdf]

nature portfolio

Corresponding author(s): Jan Terje Andersen

Last updated by author(s): Mar 24, 2025

Reporting Summary

Nature Portfolio wishes to improve the reproducibility of the work that we publish. This form provides structure for consistency and transparency in reporting. For further information on Nature Portfolio policies, see our Editorial Policies and the Editorial Policy Checklist.

Statistics

For all statistical analyses, confirm that the following items are present in the figure legend, table legend, main text, or Methods section.

n/a	Confirmed	
☐	☒	The exact sample size (*n*) for each experimental group/condition, given as a discrete number and unit of measurement
☐	☒	A statement on whether measurements were taken from distinct samples or whether the same sample was measured repeatedly
☐	☒	The statistical test(s) used AND whether they are one- or two-sided<br>*Only common tests should be described solely by name; describe more complex techniques in the Methods section.*
☒	☐	A description of all covariates tested
☐	☒	A description of any assumptions or corrections, such as tests of normality and adjustment for multiple comparisons
☐	☒	A full description of the statistical parameters including central tendency (e.g. means) or other basic estimates (e.g. regression coefficient) AND variation (e.g. standard deviation) or associated estimates of uncertainty (e.g. confidence intervals)
☐	☒	For null hypothesis testing, the test statistic (e.g. *F*, *t*, *r*) with confidence intervals, effect sizes, degrees of freedom and *P* value noted<br>*Give P values as exact values whenever suitable.*
☒	☐	For Bayesian analysis, information on the choice of priors and Markov chain Monte Carlo settings
☒	☐	For hierarchical and complex designs, identification of the appropriate level for tests and full reporting of outcomes
☒	☐	Estimates of effect sizes (e.g. Cohen's *d*, Pearson's *r*), indicating how they were calculated

Our web collection on statistics for biologists contains articles on many of the points above.

Software and code

Policy information about availability of computer code

Data collection

- Microsoft Excel; version 16.16.19 (200210) or newer
- Protein Data Bank (PDB); https://www.rcsb.org/
- Basic Local Alignment Search Tool (Nucleotide BLAST and Protein BLAST); https://blast.ncbi.nlm.nih.gov/Blast.cgi
- EMBOSS Needle Pairwise Alignment Tool; https://www.ebi.ac.uk/Tools/psa/emboss_needle/
- ClustalW2; https://www.ebi.ac.uk/Tools/msa/clustalw2/ (retired)
- Expasy Translate; https://web.expasy.org/translate/
- Expasy ProtParam tool; https://web.expasy.org/protparam/
- Magellan; version 7.2
- Unicorn; version 7.1
- Attune Cytometric Software; version 5.1.2111.1
- FACSDiva Software, version 9.3.1
- cellSens Entry; version 1.8.1
- Chromeleon Chromatography Data System Software; version 7.2.4.0
- CFX Manager Software; version 3.1.1517.0823
- GeneSys Software; version 1.4.1.0
- BioTek Gen5 Software; version 2.09
- IncuCyte S3; version 2021C
- Biacore T200 Evaluation Software; version 3.0

Data analysis	- Microsoft Excel; version 16.16.19 (200210) or newer<br>- GraphPad Prism 9 software; version 9.0.0<br>- Unicorn; version 7.1<br>- PyMOL; version 2.5.2<br>- FlowJo; version 10.8.1<br>- Winlist 3D; version 9.0.1<br>- CFX Manager software; version 3.1.1517.0823

For manuscripts utilizing custom algorithms or software that are central to the research but not yet described in published literature, software must be made available to editors and reviewers. We strongly encourage code deposition in a community repository (e.g. GitHub). See the Nature Portfolio guidelines for submitting code & software for further information.

Data

Policy information about availability of data

All manuscripts must include a data availability statement. This statement should provide the following information, where applicable:

- Accession codes, unique identifiers, or web links for publicly available datasets
- A description of any restrictions on data availability
- For clinical datasets or third party data, please ensure that the statement adheres to our policy

All data are included in the Supplementary Information or available from the authors, as are unique reagents used in this Article. The raw numbers for charts and graphs are available in the Source Data file whenever possible.
Publicly available datasets used: National Center for Biotechnology Information accession numbers: MSA: AAH49971.1, HSA: AAA98797.1.

Research involving human participants, their data, or biological material

Policy information about studies with human participants or human data. See also policy information about sex, gender (identity/presentation), and sexual orientation and race, ethnicity and racism.

Reporting on sex and gender	*Use the terms sex (biological attribute) and gender (shaped by social and cultural circumstances) carefully in order to avoid confusing both terms. Indicate if findings apply to only one sex or gender; describe whether sex and gender were considered in study design; whether sex and/or gender was determined based on self-reporting or assigned and methods used.*<br>*Provide in the source data disaggregated sex and gender data, where this information has been collected, and if consent has been obtained for sharing of individual-level data; provide overall numbers in this Reporting Summary. Please state if this information has not been collected.*<br>*Report sex- and gender-based analyses where performed, justify reasons for lack of sex- and gender-based analysis.*
Reporting on race, ethnicity, or other socially relevant groupings	*Please specify the socially constructed or socially relevant categorization variable(s) used in your manuscript and explain why they were used. Please note that such variables should not be used as proxies for other socially constructed/relevant variables (for example, race or ethnicity should not be used as a proxy for socioeconomic status).*<br>*Provide clear definitions of the relevant terms used, how they were provided (by the participants/respondents, the researchers, or third parties), and the method(s) used to classify people into the different categories (e.g. self-report, census or administrative data, social media data, etc.)*<br>*Please provide details about how you controlled for confounding variables in your analyses.*
Population characteristics	*Describe the covariate-relevant population characteristics of the human research participants (e.g. age, genotypic information, past and current diagnosis and treatment categories). If you filled out the behavioural & social sciences study design questions and have nothing to add here, write "See above."*
Recruitment	*Describe how participants were recruited. Outline any potential self-selection bias or other biases that may be present and how these are likely to impact results.*
Ethics oversight	*Identify the organization(s) that approved the study protocol.*

Note that full information on the approval of the study protocol must also be provided in the manuscript.

Field-specific reporting

Please select the one below that is the best fit for your research. If you are not sure, read the appropriate sections before making your selection.

☒ Life sciences ☐ Behavioural & social sciences ☐ Ecological, evolutionary & environmental sciences

For a reference copy of the document with all sections, see nature.com/documents/nr-reporting-summary-flat.pdf

Life sciences study design

All studies must disclose on these points even when the disclosure is negative.

Sample size	Samples sizes were determined from previous studies and publications, and are relevant for animal experiments and cellular experiments.<br>In addition, ethical perspectives was impotant in determining the sample size in animal experiments. Generally, at least 5 mice per group were

used, based on previous/pilot experiments and resulting statistical power.

Data exclusions

Data were excluded where the samples were found negative/off grid during analysis, but only where it was known to be a true error, due to positive values in repeated assays. In some cases it was not possible to repeat the whole analysis, and hence the specific samples was excluded. In the transwell assay samples were excluded where obvious leaking from one chamber to the other was detected, identified when the concentration of the opposite chamber was as high as the sample-adding chamber.

Replication

To ensure reproducability experiments were performed with two (biochemical) or three (cellular) technical replicates of each individual data point, and independent assays were run. For analysis of biological materials tests were performed twice when sufficient sample volume.
Due to ethical considerations, animal experiements were performed only once.

Randomization

Samples were, where feasable, randomized in terms of location on plate/assay. Nevertheless, in big assay set ups (mostly during flow cytometry sample preparation) the samples were systemically placed in order to prevent data mix ups.
Animals were placed into groups at a random basis, with some diviation for the experiment including male mice. In that case mice that were already housed together were used as one group.

Blinding

Investigators were partially blinded during in vitro and in vivo analyses. We saw no difference between blinded and non-blinded experiments/ analyses. Researchers performing vaccinations were not blinded. Analysis of inflammation in lung slides were fully-blinded.

Reporting for specific materials, systems and methods

We require information from authors about some types of materials, experimental systems and methods used in many studies. Here, indicate whether each material, system or method listed is relevant to your study. If you are not sure if a list item applies to your research, read the appropriate section before selecting a response.

Materials & experimental systems

n/a	Involved in the study
☐	☒ Antibodies
☐	☒ Eukaryotic cell lines
☒	☐ Palaeontology and archaeology
☐	☒ Animals and other organisms
☒	☐ Clinical data
☒	☐ Dual use research of concern
☒	☐ Plants

Methods

n/a	Involved in the study
☒	☐ ChIP-seq
☐	☒ Flow cytometry
☒	☐ MRI-based neuroimaging

Antibodies

Antibodies used

Details for antibodies used in experimental analyses (Tradename; Supplier; Reference; Lot.nr; RRID):
- Influenza A H1N1 (A/Puerto Rico/8/1934) HA ELISA pair Set; Sino Biological; SEK11684; KW09MA2601; AB_2860373 & AB_2860372
- Anti-human albumin produced in goat; Sigma-Aldrich; A1151; SLCF5233; AB_257921
- Biotinylated anti-mouse IgG1; BD Pharmingen; 553500; 0227413; AB_394885
- Biotinylated anti-mouse IgG2a; BD Pharmingen; 553502; 9140827; AB_394887
- Biotinylated anti-mouse IgG2b; BD Pharmingen; 553393; 4276786; AB_394831
- ALP-conjugated anti-mouse IgA; Mabtech; 3865-9A; 43970
- Anti-Mouse IgG (Fc specific)-Alkaline Phosphatase antibody produced in goat; Sigma; A2429; 029M4801V; AB_258000
- Anti-Human IgG (Fc specific)-Alkaline Phosphatase antibody produced in goat; Sigma; A9544; 0000083102; AB_258459
- HRP-conjugated goat anti-mouse albumin; Abcam; ab19195; GR58744-1; AB_777887
- ALP-conjugated anti-human albumin produced in goat; Bethyl Laboratories; A80-229AP; 10; AB_67020
- R-Phycoerythrin-conjugated AffiniPure Goat Anti-Human IgG; Jackson ImmunoResearch; 109-115-098; 156407; AB_2337675
- Goat anti-Mouse IgG Fc Cross Absorbed Secondary Antibody, PE; Invitrogen; 31861; VH3058083A; AB_429715
- Rat Anti-Mouse IgG1-PE; SouthernBiotech; 1144-09; F2817-W077J; AB_2794641
- Rat Anti-Mouse IgG2a-PE; SouthernBiotech; 1155-09; L5719-MJ40Z; AB_2794650
- Rat Anti-Mouse IgG2b-PE; SouthernBiotech; 1186-09; J5813-M599S; AB_2794689
- Rat Anti-Mouse IgG3-PE; SouthernBiotech; 1191-09; C0514-PI30B; AB_2794696
- Anti-Mouse IgA mAb MT45A; Mabtech; 3865-3; 2
- IgG Fraction Monoclonal Mouse Anti-Digoxin; Jackson ImmunoResearch; 200-002-156; 161187; AB_2339005
- Goat anti-mouse albumin; Abcam; ab19194; GR8434-8; AB_777886
- Mouse mAb to 6x His tag(R) [HIS.H8]; Abcam; ab18184; GR3257990-4; AB_444306
- Mouse mAb to 6x His tag(R) [HIS-1] (Alkaline Phosphatase); Abcam; ab49746; GR3347179-2; AB_867457
- Xevudy (sotrovimab); GSK. Lot no: D86G
- Evusheld (cilgavimab and tixagevimab); AstraZeneca. Lot no: CAAZ
- HRP-conjugated goat anti-mouse IgA; Bethyl; A90-103P; 56; AB_67140
- BD OptiBuild BUV395 Rat Anti-Mouse CD38 Clone 90/CD38; BD Biosciences; 740245; 3004740; AB_2739992
- BD OptiBuild BUV805 Rat Anti-Mouse CD45R/B220 Clone RA3-6B2; BD Biosciences; 748867; 2319580; AB_2873270
- BD Pharmingen Purified Hamster Anti-Mouse TCR β Chain Clone H57-597; BD Biosciences; 745846; 2271407; AB_394679
- Alexa Fluor 488 anti-mouse/human GL7 Antigen (T and B cell Activation Marker) Antibody; BioLegend; 144612; B372545;

	AB_2563285<br><br>Antibodies produced in-house (Name; batch):<br>- Anti-HA mAb (H-36-4-52); MB1. Kind gift from Siegfried Weiss, generation described in doi: 10.1084/jem.157.2.687<br>- Human IgG1 (anti-NIP) with Fc mutations (M252Y/S254T/T256E/H433K/N434F); 07/08/19. Generation described in doi: 10.4049/jimmunol.1401218<br>- Anti-mouse kappa light chain-bi mAb; Clone 187.<br>- Anti-HA mAbs for speific antigenic sites on HA; Y8-2C6, H28-E23, H17-L2, H36-11, H18-S413 and H9-A15. Kind gift from Davide Angeletti and Jonathan W. Yewdell, described in doi: 10.1038/ni.3680
Validation	All antibodies (commersial and in-house) are widely used in the laboratory and are regularly tested against in-house samples of known effect. Any abnormal results are detected, tracked and reported (to manufacturer if necessary). In addition, Citeab.com is used to check the commercial antibodies, and the CoA of commercial antibodies are checked upon delivery.

Eukaryotic cell lines

Policy information about cell lines and Sex and Gender in Research

Cell line source(s)	- HEK293E and HEK293T; ATCC<br>- Expi293F; Gibco<br>- HMEC-1-HA-FcRn-EGFP; Boston Children's Hospital, Harvard Medical School and Harvard Digestive Diseases Center, MA, USA. Generation described in doi: 10.1091/mbc.E13-04-0174<br>- MDCKII-hFcRn; pRED Roche Innovation Center Munich, Germany<br>- 293T-hACE2-TMPRSS2; Protein and Nucleic Acid Chemistry Division, Medical Research Council, Laboratory of Molecular Biology, Cambridge, United Kingdom. Generation described in doi: 10.1371/journal.ppat.1009246<br>- HeLa-ACE2; Department of Immunology and Microbiology, The Scripps Research Institute, and Division of Infectious Diseases, Department of Medicine, University of California, La Jolla, CA, USA. Generation described in doi: 10.1126/science.abc7520
Authentication	Authentication of all cell lines: visual inspection of morphology, proliferation rate monitored.<br>- HEK293E and Expi293F; protein production rates of in-house standardized antibody variants monitored.<br>- HMEC-1-HA-FcRn-EGFP; FcRn expression validated by EGFP expression on FACS.<br>- MDCK-hFcRn; polarization rate and level monitored; transcellular transport of FcRn-negative antibody monitored.
Mycoplasma contamination	Cell lines tested negative for mycoplasma contamination.
Commonly misidentified lines<br>(See ICLAC register)	No commonly misidentified cell lines were used in the study.

Animals and other research organisms

Policy information about studies involving animals; ARRIVE guidelines recommended for reporting animal research, and Sex and Gender in Research

Laboratory animals	Laboratory animals:<br>Mice of the following strains:<br>- Balb/C, females age 6-8 weeks, 5-12 mice/group, or females age 8-9 weeks 5-6 mice/group<br>- Tg32-hFc (B6.Cg-Tg(FCGRT)32Dcr Fcgrttm1Dcr Ighg1em2(IGHG1)Mvw/MvwJ), males and females age 6-13 weeks, 3-7 mice/group, or females age 31 weeks, 5-6 mice/group<br>- HSA/hFcRn (Fcrgttm1.1(FCRGT)Geno;Albtm1.1(ALB)Geno), females age 13-14 weeks, 6 mice/group<br>- K18-hACE2 (B6.Cg-Tg(K18-ACE2)2Prlmn/J), females age 10-12 weeks, 10-12 mice/group
Wild animals	The study did not include wild animals.
Reporting on sex	Both male and female mice were used and findings apply to both sex. More female mice were used due to the convenience of housing and randomization of groups, as well as the possibility to harvest vaginal flushes for detection of antibodies at distal mucsal sites.
Field-collected samples	The study did not include field-collected samples.
Ethics oversight	The animal studies were carried out at The Department for Comparative Medicine, Oslo University Hospital (Rikshospitalet, Oslo, Norway) in accordance with the Guide for the Care and Use of Laboratory Animals of the Norwegian National Institute of Health, and the experiments were approved by The Norwegian Food Safety Authority, or at The Scripps Research Institute (La Jolla, CA, USA) in accordance with IACUC approved procedures.

Note that full information on the approval of the study protocol must also be provided in the manuscript.

Plants

Seed stocks

Report on the source of all seed stocks or other plant material used. If applicable, state the seed stock centre and catalogue number. If plant specimens were collected from the field, describe the collection location, date and sampling procedures.

Novel plant genotypes

Describe the methods by which all novel plant genotypes were produced. This includes those generated by transgenic approaches, gene editing, chemical/radiation-based mutagenesis and hybridization. For transgenic lines, describe the transformation method, the number of independent lines analyzed and the generation upon which experiments were performed. For gene-edited lines, describe the editor used, the endogenous sequence targeted for editing, the targeting guide RNA sequence (if applicable) and how the editor was applied.

Authentication

Describe any authentication procedures for each seed stock used or novel genotype generated. Describe any experiments used to assess the effect of a mutation and, where applicable, how potential secondary effects (e.g. second site T-DNA insertions, mosiacism, off-target gene editing) were examined.

Flow Cytometry

Plots

Confirm that:

- [ ] The axis labels state the marker and fluorochrome used (e.g. CD4-FITC).
- [x] The axis scales are clearly visible. Include numbers along axes only for bottom left plot of group (a 'group' is an analysis of identical markers).
- [x] All plots are contour plots with outliers or pseudocolor plots.
- [ ] A numerical value for number of cells or percentage (with statistics) is provided.

Methodology

Sample preparation	Sera were diluted in assay buffer as specified under "Materials and Methods" and incubated with bead based arrays.<br>For flow cytometry experimets on mediastinal lymph nodes, the harvested lymph nodes were pooled within each treatment group, mashed and stained as described under "Materials and Methods".
Instrument	Attune NxT Acoustic Focusing Cytometer, Invitrogen (serological analyses and lymph node analyses)<br>FACSymphony A5, BD Bioscience (lymph nodes analyses)
Software	Serological analyses: Data collection: Attune Cytometric Software, Invitrogen. Analysis: Winlist 3D, Verity Software House<br>Lymph node analyses: Data collection: FACSDiva Software, BD Bioscience or Attune Cytometric Software, Invitrogen. Analysis: FlowJo, BD Bioscience.
Cell population abundance	NA
Gating strategy	Serological analyses: FSC/SSC: Gating of single beads. Flourecent bar codes gating on: Bodipy, Cy5, Pacific Blue.<br>Lymph nodes analyses: Gated on single cells, viable cells, TCRbneg, CD19pos, B220pos, CD38neg, GL7pos, Agpos OR single cells, viable cells, TCRbneg, B220pos, CD38neg, GL7pos, Agpos.

- [x] Tick this box to confirm that a figure exemplifying the gating strategy is provided in the Supplementary Information.
